# Mutations in *PmUFGT3* contribute to color variation of fruit skin in Japanese apricot (*Prunus mume* Sieb. et Zucc.)

**DOI:** 10.1186/s12870-022-03693-8

**Published:** 2022-06-24

**Authors:** Xiaopeng Ni, Zhaojun Ni, Kenneth Omondi Ouma, Zhihong Gao

**Affiliations:** College of Horticulture, Nanjing Agricultural University, No. 1 Weigang, Nanjing, 210095 China

**Keywords:** *Prunus mume*, Anthocyanin, UFGT, SNP, Mutation

## Abstract

**Background:**

Japanese apricot (*Prunus mume* Sieb. et Zucc.) is popular for both ornamental and processing value, fruit color affects the processing quality, and red pigmentation is the most obvious phenotype associated with fruit color variation in Japanese apricot, mutations in structural genes in the anthocyanin pathway can disrupt the red pigmentation, while the formation mechanism of the red color trait in Japanese apricot is still unclear.

**Results:**

One SNP marker (PmuSNP_27) located within *PmUFGT3* gene coding region was found highly polymorphic among 44 different fruit skin color cultivars and relative to anthocyanin biosynthesis in Japanese apricot. Meantime, critical mutations were identified in two alleles of *PmUFGT3* in the green-skinned type is inactivated by seven nonsense mutations in the coding region, which leads to seven amino acid substitution, resulting in an inactive UFGT enzyme. Overexpression of the *PmUFGT3* allele from red-skinned Japanese apricot in green-skinned fruit lines resulted in greater anthocyanin accumulation in fruit skin. Expression of same allele in an Arabidopsis T-DNA mutant deficient in anthocyanidin activity the accumulation of anthocyanins. In addition, using site-directed mutagenesis, we created a single-base substitution mutation (G to T) of *PmUFGT3* isolated from green-skinned cultivar*,* which caused an E to D amino acid substitution and restored the function of the inactive allele of *PmUFGT3* from a green-skinned individual.

**Conclusion:**

This study confirms the function of *PmUFGT3,* and provides insight into the mechanism underlying fruit color determination in Japanese apricot, and possible approaches towards genetic engineering of fruit color.

**Supplementary Information:**

The online version contains supplementary material available at 10.1186/s12870-022-03693-8.

## Background

Japanese apricot (*Prunus mume* Sieb. et Zucc.), an ornamental plant and fruit tree originating from Southwest China and is widely cultivated in all of East Asia and Japan. According to its various applications, Japanese apricot is divided into two groups, i.e. fruiting-mei and flowering-mei. Fruiting-mei has been cultivated and consumed in China for more than 7000 years. China, being the origin of Japanese apricot, is rich in good quality germplasm, and approximately 190 fruiting cultivars have been recorded [1]. The fruit is usually processed into many value-added products, including salted-mei, mei wine, and juice, and is considered to have high nutritional and medicinal value [2]. Fruit colour works as a significant factor to evaluate fruit value and quality, and attracts customer’s attention to increase sales [3].

Plentiful fruit colors result from the accumulation of various anthocyanins [4], which are the main water-soluble pigments belong to parent class of flavonoids [5], synthesized through the phenylpropanoid pathway [6], accumulated and localized in vacuoles in different plant tissues such as fruit, flowers, leaves, roots, stems [7–9]. The accumulation of anthocyanins leads to multiple characteristics: red, purple or blue hue which depends on pH of the vacuole, not only improve ornamental value of merchandise, but also has physiological healthy benefits on several disease, Alzheimer [10], Parkinson [11], diabetes [12], and has anticancer importance [13].

The anthocyanins biosynthetic pathway has been widely reported in various fruits, including grape [14], apple [15], peach [16], pear [17] and plum [18]. The phenylpropanoid and flavonoid pathway involve in the biosynthesis of anthocyanins, several essential structural genes encode enzymes such as cinnamic acid 4-hydroxylase (C4H), phenylalanine ammonia-lyase (PAL) and 4-coumarate-CoA ligase (4CL), chalcone isomerase (CHI), chalcone synthase (CHS), dihydroflavonol 4-reductase (DFR), flavanone 3-hydroxylase (F3H), flavanone 3’-hydroxylase (F3’H), leucoanthocyanidin dioxygenase (LDOX) and UDP-glucose flavonoid- 3-O-glycosyltransferase (UFGT) participated in regulation process [19]. *UFGT* works as the final structural gene to catalyze the 3-O-glucosylation of anthocyanidins in the biosynthesis process [20, 21]. *UFGT* was only been detected and expressed in red grapes, and it is not found in white grapes which appear to lack anthocyanins, even same as other different tissues [14]. *UFGT* expression was consistent with accumulation of anthocyanins and flavonols in the pericarp of litchi, and plays as a key factor to influence the anthocyanins biosynthesis and fruit color in litchi [22], *AcUFGT3* is found as the key gene to regulate the biosynthesis and accumulation of anthocyanins in red-fleshed ‘Hongyang’ kiwifruit [23].

Mutations in structural genes in the anthocyanin pathway can disrupt the red floral pigmentation [24]. Both no-sense and mis-sense mutations in the chalcone flavonone isomerase (*Cfi*) resulting in the absence of anthocyanin in barley and rice [25]. Mutations in the sequence of dihydroflavonol 4-reductase resulted in the absence of anthocyanins and proanthocyanins of barley [26]. One single-base deletion in the flavanone 3’-hydroxylase which represents the *T* gene of soybean controls gray pubescence color [27]. Frameshift mutation was characterized in the UDP-glucose flavonoid-3-O-glycosyltransferase in Japanese (*Ipomoea nil*) and the common (*I. purpurea*) morning glory which caused about 80% reduction of anthocyanin accumulation, such defects cause pale flower pigmentation [28]. Although these studies have provided valuable insights into the mechanism of coloration, the regulatory mechanism of anthocyanin biosynthesis and the cause for fruit color difference between two lines in Japanese apricot remain unclear.

In recent years, molecular markers developed based on resequencing data have received extensive attention and research, especially the third generation of DNA molecular markers represented by single nucleotide polymorphisms (SNPs), which are abundant in plant genomes and widely recognized as a large number, genetically stable and easily detectable high-efficiency molecular marker, with an estimated more than one SNP per thousand bases [29], and is the most common type of genomic variation among different individuals within a species [30]. SNP molecular markers have been applied in a variety of research areas in Japanese apricot, such as germplasm resource identification [31, 32], genetic evolutionary analysis [33, 34], genetic map construction [35, 36] and flower color gene localization [37, 38] et al. Fang et al. identified 95 SNPs among 67 reliable sequences in 13 homologous sequence groups by amplifying fragment length polymorphism fragments (AFLP) of several varieties, and the high quality and low error rate of the repeat sequencing results demonstrated that the development of SNP molecular markers based on AFLP of Japanese apricot could be used to identify Chinese and Japanese germplasm resources [31]. Li et al. investigated a total of 68 Japanese apricot cultivars genetic relationships between two categories of flowering and fruiting mei cultivars. 92 SNPs were detected from the DNA of all cultivars by nine pairs of PCR primers with a distribution frequency of one SNP per 32 bp. Using these SNPs, mei cultivars were clustered and analyzed into 13 groups, different from the original classification of flowering and fruiting group, proving that fruiting and flowering mei cultivars are genetically close to each other, indicating that SNP molecular markers are an effective tool to reveal the genetic evolutionary analysis of Japanese apricot [33]. Kitamura et al. developed the first high-density SNP genetic map of Japanese apricot based on genotyping-by-sequencing (GBS) technology, and used parents with different cooling requirements and their hybrid progeny to analyze the phenotype of dormancy-related traits. *PmDAM6* was identified by quantitative trait locus (QTL) analysis as a dose-dependent inhibitor of dormancy breaking to control bud dormancy of Japanese apricot [36]. Zhang et al. identified 10 floral trait QTLs and genomic regions including petal color, stigma color, calyx color, and bud color by genome-wide association study (GWAS), and found that *MYB108* may played a key role in regulating the genetic control and evolution of petal color in *Prunus* [38], but molecular markers related to fruit skin color in Japanese apricot have been reported.

In this study, we investigated the mechanism underlying fruit skin color variation in Japanese apricot. Recently, a cDNA coding the UDP-glycose: flavonoid- 3-O-glycosyltransferase gene (*PmUFGT3*) was isolated from fruit skin from a red-skinned variety and shown to function in anthocyanin biosynthesis in vitro, and by complementation of a gene mutation in Arabidopsis. Meantime, according to resequencing data of previous study [39], one key nonsense point mutation on chromesome 4 linked to *PmUFGT3* was found and worked as highly polymorphic PmuSNP_27 SNP molecular maker, transversion between T and G at bp1332 in the CDS resulting in one amino acid substitution D to E in green-skinned fruits of Japanese apricot compared with red-skinned cultivars, and propose a working model for *PmUFGT3* to modify cyanidin 3-O-glucoside in Japanese apricot.

## Results

### Fruit skin color variation in Japanese apricot

**Fig. 1:**
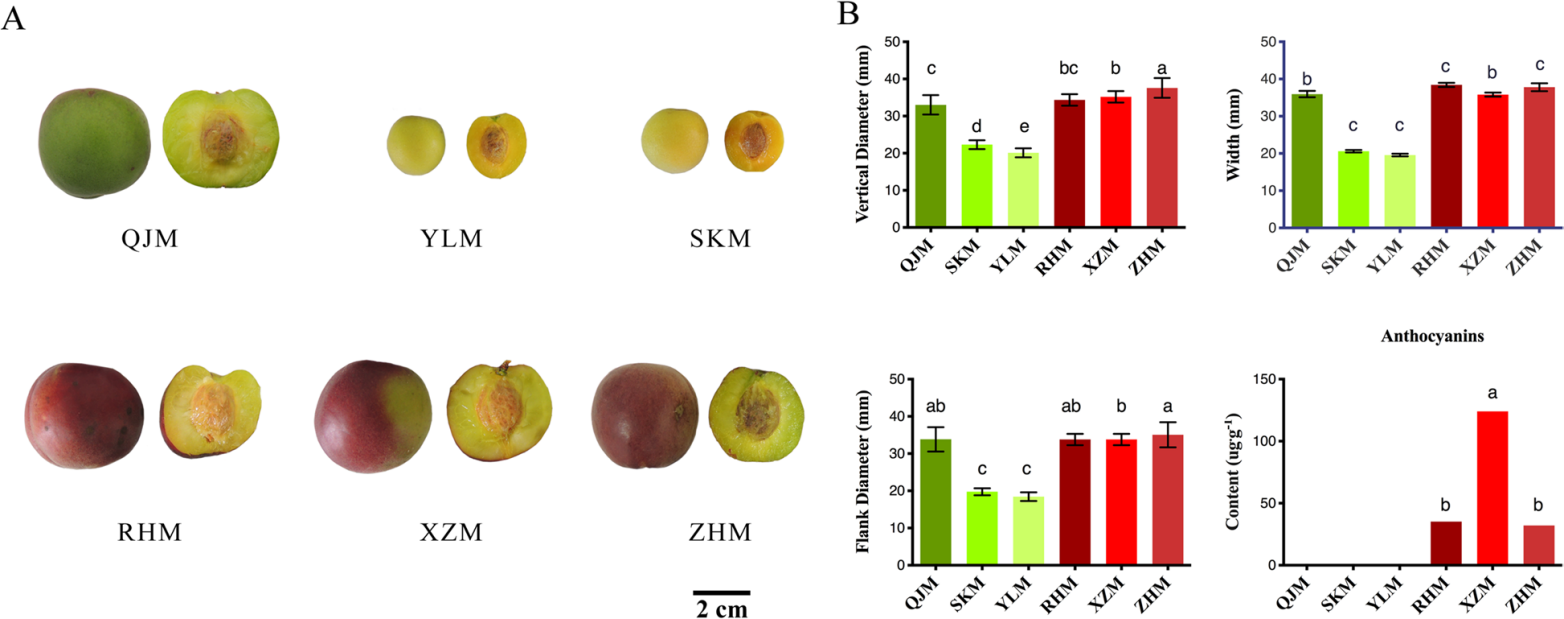
Fruits of the six Japanese apricot cultivars used in this study. **A** The top row shows fruit from the three green-skinned cultivars; the lower row show fruits from the three red-skinned cultivars. **B** Flank diameter, vertical diameter, width and anthocyanins context of six Japanese apricot cultivars. Different letters above the bars indicate significantly different mean values

The red-skinned varieties were significantly distinguished from the green-skinned varieties due to the red pigmentation of the epidermis by observation of fruit skin phenotype (Fig. 1A). ‘XZM’ was the cultivar with the largest coloring area and the highest anthocyanin content, which was significantly higher than ‘RHM’ and ‘ZHM’ these two red-skinned cultivars, while there were no anthocyanins detected in all green-skinned cultivars. Based on phenotypic indicators of flank diameter, vertical diameter and width, green-skinned cultivar ‘QJM’ and all red-skinned cultivar showed no significant difference in fruit size, but the other two green-skinned cultivars, ‘SKM’ and ‘YLM’ were significantly lower than these four cultivars (Fig. 1B).

### Anthocyanin content between red- and green-skinned fruits

In terms of anthocyanin content, red-skinned fruits contained high levels while no anthocyanin was detected in green-skinned fruits (Fig. 1B). Fruit from the ‘XZM’ cultivar exhibited the highest anthocyanins content, more than two folds higher than the other two red-skinned cultivars ‘RHM’ and ‘ZHM’.

### Genetic diversity

The number of effective alleles is the reciprocal of gene homozygosity, which is an indicator of population genetic variation, which indicates the degree of distribution in the allele population. For *PmUFGT3* gene, there was one non-synonymous SNP (PmuSNP_27) in the CDS at bp1332 (T/G), two different fruit skin groups (green and red-skinned) and total 44 accessions of Japanese apricot cultivars were used for this SNP validation, there were total 88 alleles among 44 varieties, average two alleles per SNP site of each cultivar. The number of observed alleles and effective alleles of PmuSNP_27 were 2 and 1.97, respectively (Table 1).

**Table 1:** Parameter values of PmuSNP_27 SNP polymorphism in 44 Japanese apricot genotypes

Markers	*Na*	*Ne*	*I*	*He*	*Fst*	*Nm*	*PIC*
PmuSNP_27	2	1.97	0.69	0.49	0.6	0.16	0.581

*Na* Observed number of alleles, *Ne* Effective number of alleles, *I* Shannon’s information index, *He* Nei’s gene diversity, *Fst* Fixation index, *Nm*: Gene flow, *PIC* Polymorphism information content

Shannon’s information index (*I*) of PmuSNP_27 was 0.69, Nei’s gene diversity (*He*) was 0.49, gene flow (*Nm*) is 0.16. Fixation index (*Fst*) of PmuSNP_27 was 0.6, which indicated that there is significant genetic difference between green and red-skinned cultivars group. More importantly, polymorphic information content (*PIC*) is an indicator of the diversity of species. *PIC* > 0.5 in the population indicates that the site is highly polymorphic. In this study, PmuSNP_27 was highly polymorphic (0.581). PmuSNP_27 was highly congruent relative to identifying genotypes and for estimating population genetic differences, meanwhile marker PmuSNP_27 (*PmUFGT3****)*** was also the candidate gene involved in the biosynthesis of anthocyanin, suggesting that it is the potential vital regulatory gene for the biosynthesis of anthocyanin in Japanese apricot.

### Isolation of full-length *PmUFGT3* cDNAs

The full length of the *PmUFGT3* gene sequence of both red- and green-skinned ‘RHM’ and ‘QJM’ fruits was 1509 bp. This sequence was submitted to GenBank under the accession number xxx (uploading). A comparison of the full-length genomic sequence of *PmUFGT3* with that of the cDNA isolated from the corresponding apricot fruits revealed one exons of 1509 bp, without introns. It shared 99.8% homology with the *PmUFGT3* gene of *Prunus mume* (GenBank accession number 103328988).

**Fig. 2:**
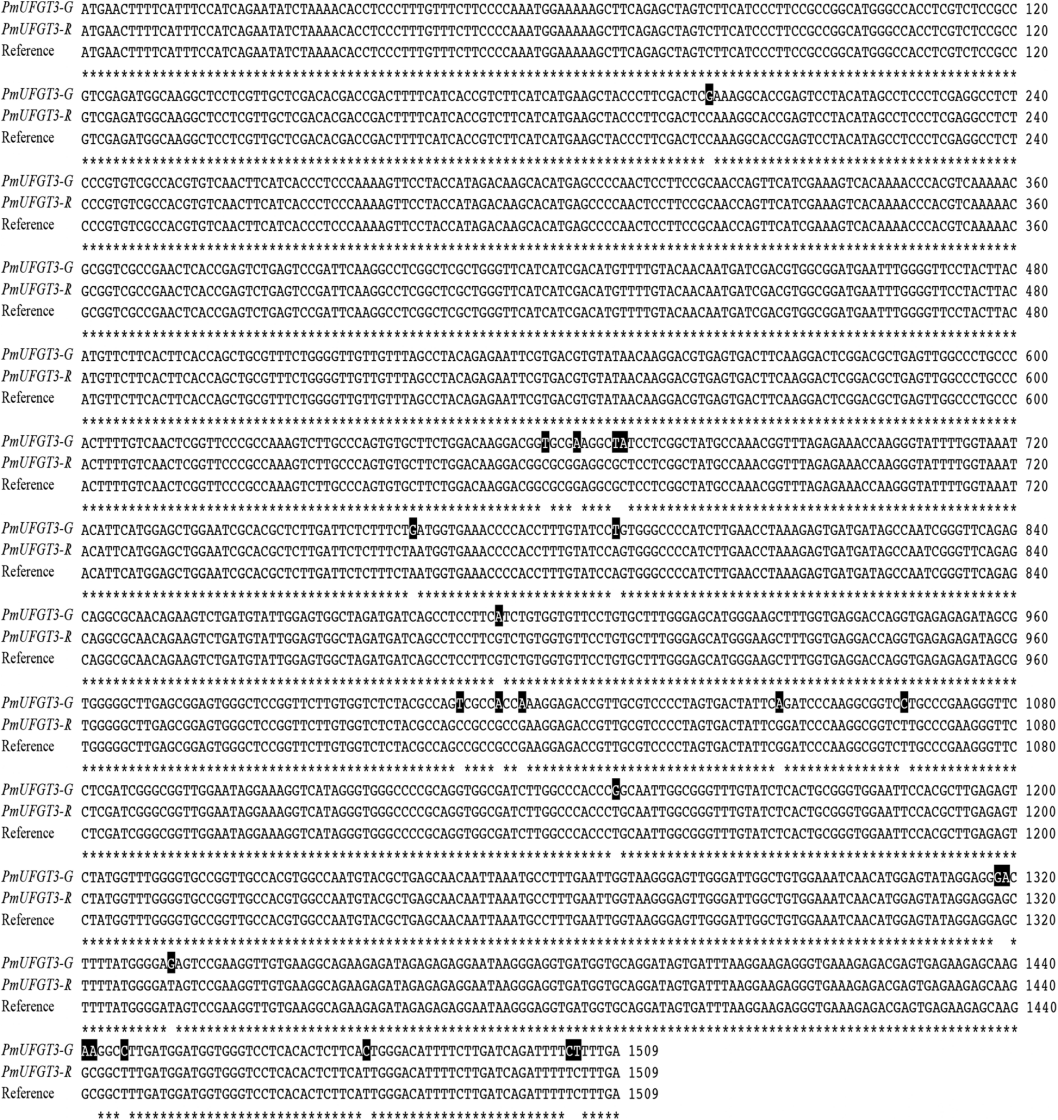
Multiple sequence alignments of nucleotide sequences of *PmUFGT3* from red and green-skinned fruits of Japanese apricot

A total of 23 SNPs were identified by comparing *PmUFGT3* cDNA sequences between red and green-skinned fruits of Japanese apricots (Fig. 2). Among them, transition substitutions (69.6%) were more frequent than transversions (30.4%). Among the base transitions, 9 transitions between G and A were higher than 7 transitions between C and T. For the transversions, the substitutions were as follows (in decreasing order of frequency): 3 times for T and G, including the PmuSNP_27 site located at bp1332 (T/G), 2 times for A and G, and 1 time for both G and C with T and A.

### Characterization of *pmUFGT3* cDNA and the encoded protein

The SNPs at positions 664, 669–670, 764, 1014, 1318–1319, 1332, 1441–1442 bp were classified as seven nonsense mutations, resulting in seven amino acid substitution in green-skinned fruits of Japanese apricot, while SNPs at the 13 other positions were classified as synonymous mutations and did not affect the protein sequence.

**Fig. 3:**
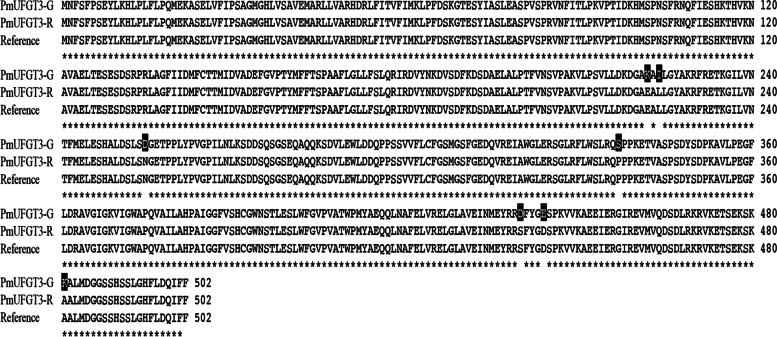
Multiple sequence alignments of amino acid sequences of PmUFGT3 between red and green-skinned fruits of Japanese apricot using the DNAMAN program. The M in the leader sequences are underlined while stop codons are marked with an F

The full-length cDNA of *pmUFGT3* from red fruits of Japanese apricot had a length of 1509 bp, encoding a polypeptide of approximately 502 amino acid residues, which corresponds to a molecular mass of 55.82 kDa, with a theoretical pI of 5.1 (Fig. 3). An analysis of the secondary structure of the predicted *PmUFGT3* protein revealed that all examined *PmUFGT3* contained a Glycosyltransferase_GTB-type super family binding domain (accession: cl10013) (Additional file 1). Furthermore, secondary structure analysis of the predicted *PmUFGT3* revealed that this predominantly consisted of 216 alpha-helices (43.03%), followed by 190 random coils (37.85%), 72 extended strand (14.34%) and 24 beta turn (4.78%) (Additional file 2).

**Fig. 4:**
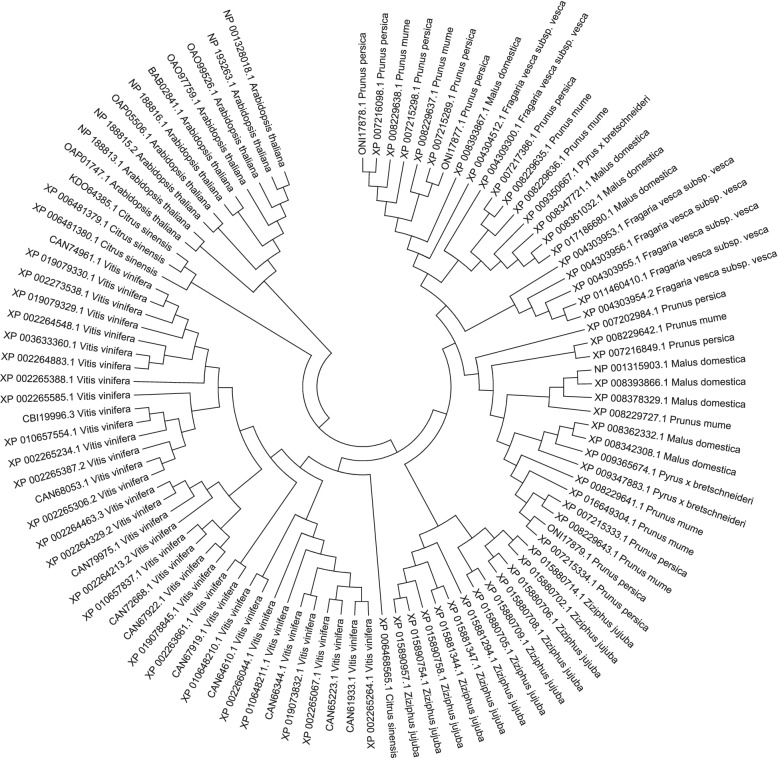
Unrooted phylogenetic tree of *PmUFGT3* (GenBank accessions: XP008229637.1) and flavonoid glycosyltransferase amino acid sequences. The number adjacent branches indicate maximum parsimony bootstrap values for the corresponding node. The scale bar indicates the number of differences per 100 residues derived from the ClustalW alignment. The phylogenetic tree was generated using TreeView software

The phylogenetic tree based on the amino acid sequences of PmUFGT3 in red fruits of Japanese apricot and other nucleic acid sequences of UGTs of various plant species are shown in Fig. 4. Although the in vitro substrate specificities and in vivo functions of flavonoid UGTs cannot be accurately predicted using amino acid sequences alone (Fig. 4). Amino acid sequences of PmUFGT3 (GenBank accessions: XP008229637.1) has close relationship with *Prunus persica* and *Malus domestica.*

### Construction of the plant expression vector and transient transformation of Japanese apricot skins

To confirm that *PmUFGT3* regulates anthocyanin biosynthesis in Japanese apricot, a transient assay of anthocyanin production was primarily developed in immature Japanese apricot. The full-length *PmUFGT3* cDNA was expressed under the control of the constitutive cauliflower mosaic virus 35S promoter in pCAMBIA1301, injection of *A. tumefaciens* trains (*GV3101*) in Japanese apricot fruit.

**Fig. 5:**
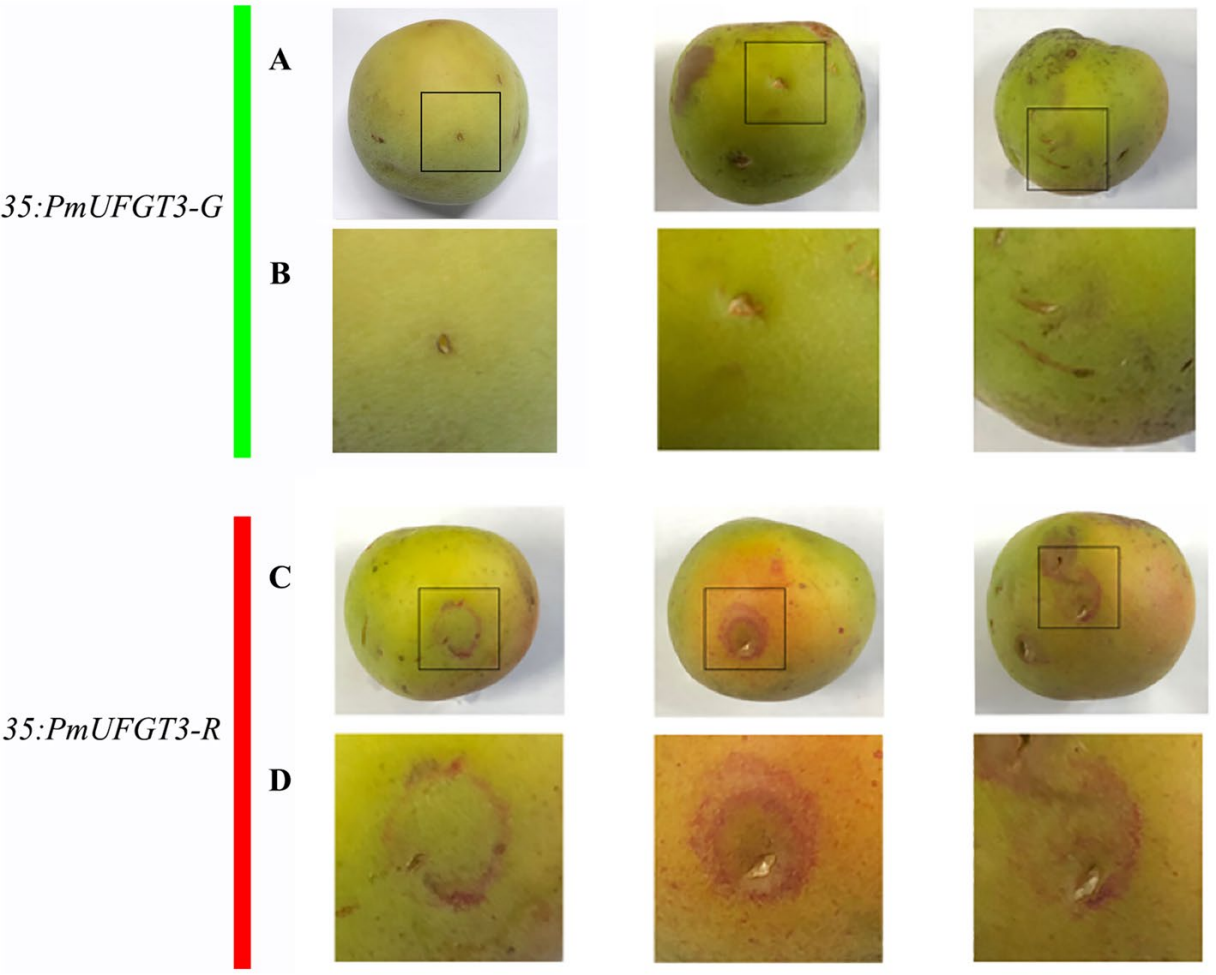
Transient expression of *PmUFGT3* in Japanese apricot fruit skin

As can be seen from Fig. 5, transient expression of *PmUFGT3* from red-skinned cultivars ‘RHM’, showed red coloration around the injection site of immature ‘QJM’ fruit skin by transient expression in the shape of a circle, however, green-skinned cultivars ‘QJM’ *PmUFGT3* did not show red coloration around the injection site of immature ‘QJM’ fruit skin. The results showed that the *PmUFGT3* gene of red-skinned *PmUFGT3-R* could promote the synthesis and accumulation of anthocyanin in the peel of ‘QJM’ fruit skin, while the green-skinned *PmUFGT3-G* gene could not promote the synthesis and accumulation of anthocyanin in the fruit skin of ‘QJM’.

**Fig. 6:**
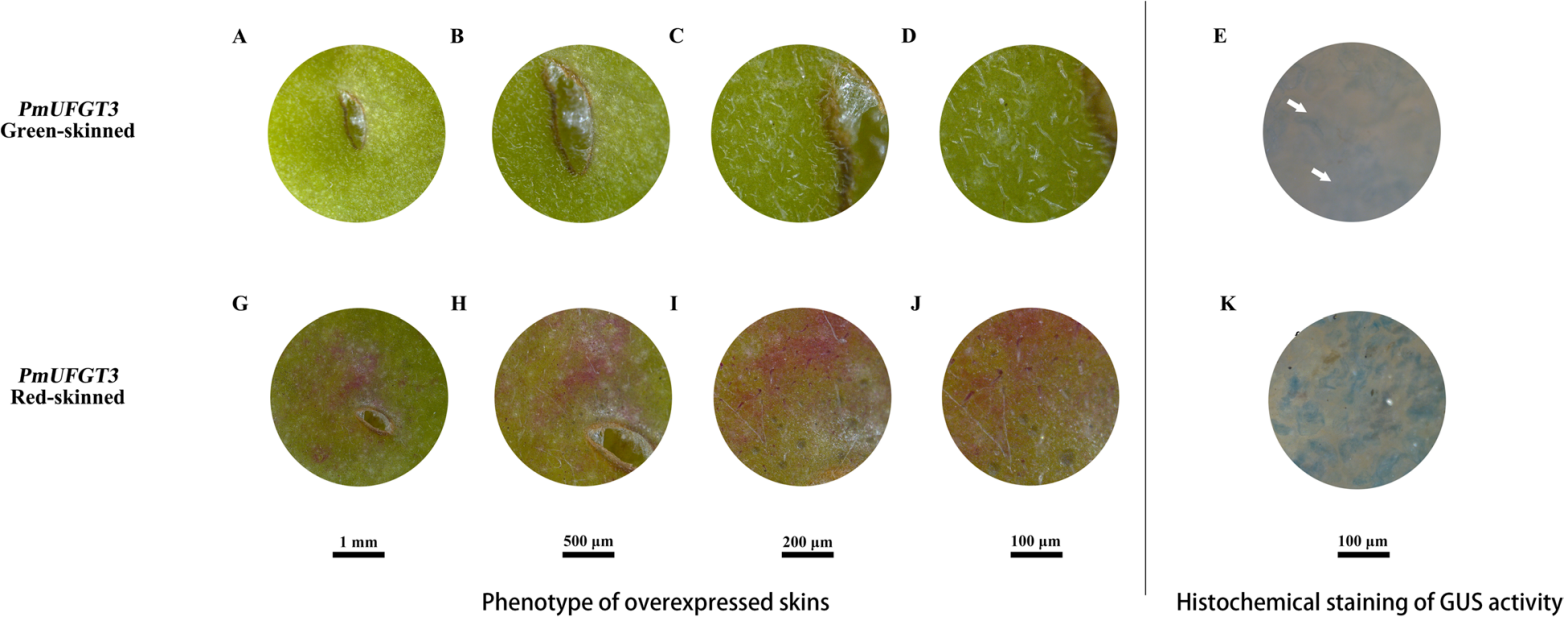
Transient expression of *PmUFGT3* in Japanese apricot fruit skin and GUS activity

The fine structure of transient expression of green and red-skinned *PmUFGT3* in immature green-skinned ‘QJM’ pericarp was observed by somatic microscopy (Fig. 6). There was no significant difference in the brown wounds caused by *PmUFGT3* at the injection site, while red-skinned *PmUFGT3-R* transient expression around the injection hole had a ring of red coloration, which is dark red in color and also accumulates red within the white villi. In contrast, the epidermal microstructure of red-skinned *PmUFGT3-G* did not have any changes.

GUS expression activity was detected in both green and red-skinned *PmUFGT3* transient expression in fruit skin, and most of the green-skinned *PmUFGT3* transient GUS expression was on the cell membrane with low activity. The red-skinned *PmUFGT3* transient GUS expression was found in both cell membrane and cytoplasm and was more active. The results showed that red-skinned *PmUFGT3* transient expression functioned normally as UFGT, while green-skinned *PmUFGT3* transient expression was slightly weaker (Fig. 6).

### Transient transformation analysis in Arabidopsis mutant line *Atufgt*

**Fig. 7:**
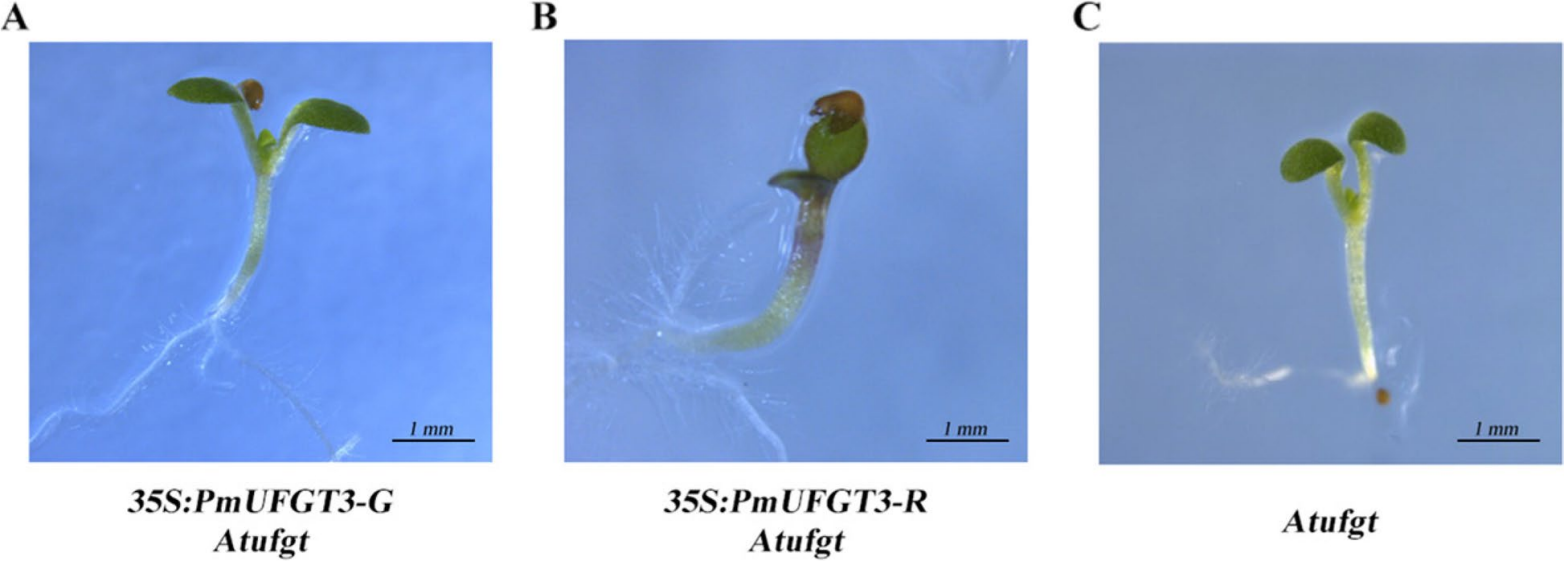
Overexpression of *PmUFGT3* in Arabidopsis mutant line *Atufgt*

To confirm that *PmUFGT3* regulates anthocyanin biosynthesis in Japanese apricot, the full-length *PmUFGT3* cDNA was expressed under the control of the constitutive cauliflower mosaic virus 35S promoter in the *UFGT* mutant, *Atufgt*. A total of ten independent transgenic plants were obtained and verified for transgene integration by PCR using primers designed to detect the binary vector pCAMBIA1301. The T2 transgenic Arabidopsis plant injected by *PmUFGT3* from red-skinned cultivars showed complementation of the red-skinned phenotype, determined by visual examination of the hypocotyl color (Fig. 7). While the transgenic plant injected by *PmUFGT3* from green-skinned cultivars showed complementation of the green-skinned phenotype, determined by visual examination of the hypocotyl color (Fig. 7), no color pigmentation compare with red-skinned *PmUFGT3*. Anthocyanins were detected in leaves and hypocotyl edonary axis from red-skinned, which without SNP mutation induce anthocyanin accumulation in Arabidopsis thaliana leave. *PmUFGT3* is a galactosyltransferase, catalyzing the glycosylation of cyanidin and used the UDP-galactose more efficiently than UDP-glucose.

### Point mutation at the conserved *UFGT* domain-encoding region abolishes the function of *PmUFGT3* at an early stage of anthocyanin accumulation

The *PmUFGT3-GM1*, *PmUFGT3-GM2*, *PmUFGT3-GM3*, *PmUFGT3-GM4*, *PmUFGT3-GM5*, *PmUFGT3*-*GM6*, and *PmUFGT3*-*GM7* mutations were generated by target primers, which were designed according to the instructions in the site-directed mutagenesis kit. In additional file 3, codon D222 (AAG) was replaced by CAG (E) for *PmUFGT3-GM1*, codon D224 (ATC) was changed to CTC (L) for *PmUFGT3-GM2*, codon D255 (GAT) was replaced by AAT (N) for *PmUFGT3-GM3*, codon D338 (TCG) was changed to CCG (P) for *PmUFGT3-GM4*, codon D440 (GAC) was replaced by AGC (S) for *PmUFGT3-GM5*, codon D444 (GAG) was changed to GAT (D) for *PmUFGT3-GM6*, and the codon D481 (AAG) was substituted with GCG (A) for *PmUFGT3-GM7*.

**Fig. 8:**
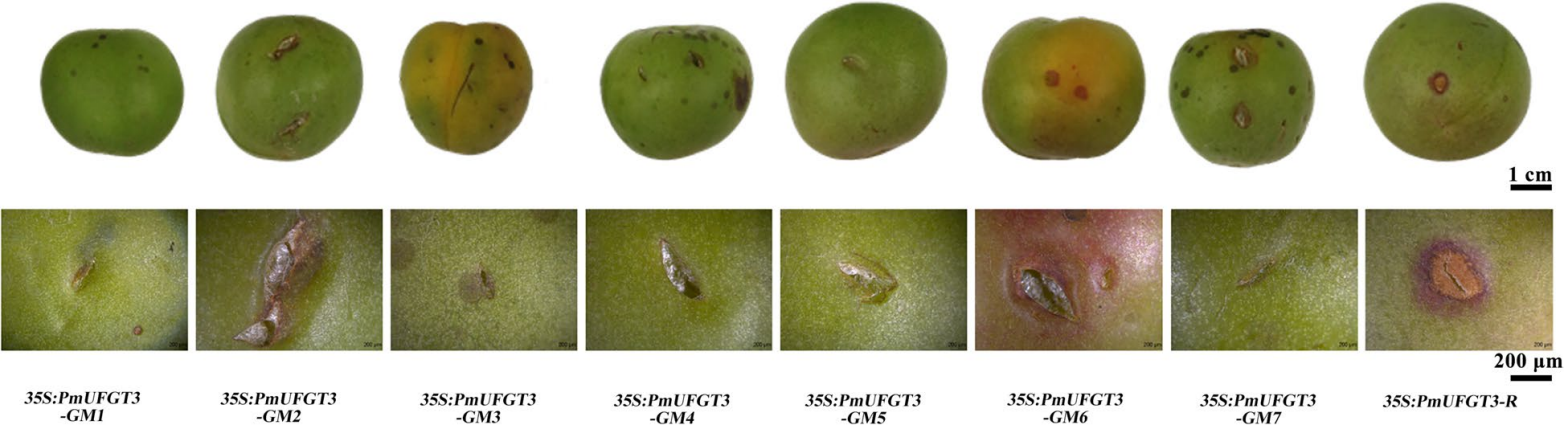
Transient expression of *PmUFGT3* site-directed mutagenesis in Japanese apricot fruit skin

The transient expression of seven site-directed mutagenesis *PmUFGT3* from green-skinned cultivars ‘QJM’, which only one *PmUFGT3-GM6* mutagenesis induced the formation of red cells in the skin, the transgenic plant showed complementation of the red-skinned phenotype as *PmUFGT3-R* from red-skinned cultivars ‘RHM’, determined by visual examination of the skin color (Fig. 8) while other six site-directed mutagenesis *PmUFGT3* still have the same phenotype as green-skinned cultivars which no red-pigmentation on the fruit skin.

### The different position of *PmUFGT3* of Green-skinned fruit expression in Japanese apricot and Arabidopsis thaliana *Atufgt*

**Fig. 9:**
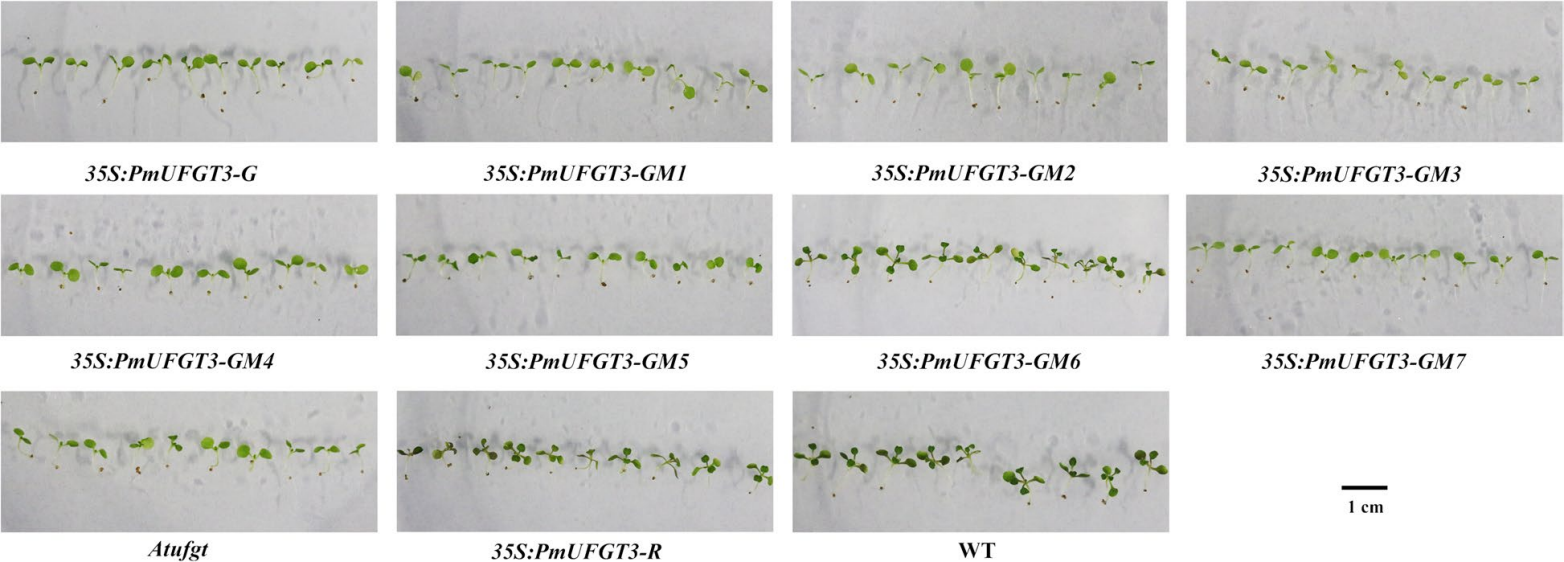
Overexpression of *PmUFGT3* site-directed mutagenesis in Arabidopsis mutant line *Atufgt*

**Fig. 10:**
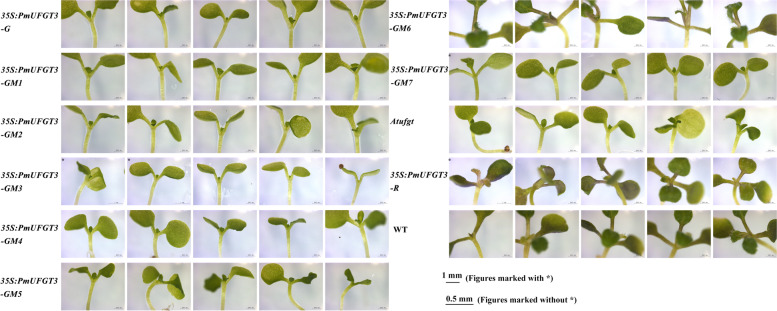
Overexpression of *PmUFGT3* site-directed mutagenesis in Arabidopsis mutant line *Atufgt* (hypocotyl)

To further confirm that *PmUFGT3* regulates anthocyanin biosynthesis in Japanese apricot, the full-length seven site-directed mutagenesis *PmUFGT3* from green-skinned cultivars ‘QJM’ was expressed under the control of the constitutive cauliflower mosaic virus 35S promoter in the *UFGT* mutant, *Atufgt*. A total of ten independent transgenic plants were obtained and verified for transgene integration by PCR using primers designed to detect the binary vector pCAMBIA1301. The transgenic plant injected by *PmUFGT3-GM6* mutagenesis showed complementation of the red-skinned phenotype as *PmUFGT3-R* from red-skinned cultivars ‘RHM’, determined by visual examination of the red hypocotyl color (Fig. 9 and 10). While the transgenic plant injected by other six *PmUFGT3-GM1* to *GM5* and *GM7* from green-skinned cultivars showed complementation of the green-skinned phenotype determined by visual examination of the green hypocotyl color (Fig. 10), no color pigmentation compare with red-skinned *PmUFGT3-R*. Anthocyanins were detected in leaves and hypocotyl edonary axis from red-skinned, which site-directed mutagenesis mutation of codon D444 (GAG) was changed to GAT (D) for *PmUFGT3-GM6* induce anthocyanin accumulation in Arabidopsis thaliana hypocotyl.

## Discussion

Non-synonymous mutations occurring in the coding region of *UFGT*, a structural gene that catalyzes the final step of anthocyanin biosynthesis, have been shown to affect anthocyanin synthesis in a variety of plants [40, 41], yet the function of *UFGT* has still not been reported in Japanese apricot. The objectives of this study were twofold: firstly, to confirm that *UFGT* is a key gene for anthocyanin synthesis in Japanese apricot fruit skin through transient expression and Arabidopsis mutant complementation experiments; Secondly, to explore the key mutational SNPs that lead to UFGT dysfunction in anthocyanin biosynthesis by targeted mutagenesis, which will provide a basis for future analysis of anthocyanin accumulation in Japanese apricot.

### *PmUFGT3* is the key gene and encoded enzyme related to anthocyanin accumulation

The recessive mutations of UDP-glucose: flavonol glucosyltransferase at the *bronze* locus were firstly reported and cloned using the transposable controlling element *Activator* (Ac) from maize in 1984 [42], which conducted the study of UFGT. Since then, UFGT enzymes were confirmed using uridine diphosphate-D-glucose (UDPG) as glucosyl-donor to catalyse the glucosylation of cyanidin and isolated and purified from red cabbage (*Brassica oleracea)* [43] and *Haplopappus gracilis* (Nutt.) [44], followed by soybean (*Glycine max L.*) [45] and red campion (*Silene dioica*) [46], those studies can validate the red color pigmentation depends on the function of UFGT. At the molecular level, the full length cDNA of *UFGT* was cloned to understand the molecular biology and biochemistry of anthocyanins biosynthesis from *Gentiana trifloral* [47]*.* In particular, its important role in fruits has been valued, UFGT was reported as an enzyme responsible for a late step of anthocyanin biosynthesis in grapevive (*Vitis vinifera*), to glucosylate anthocyanidins in red fruit during ripening [48]. The biosynthesis of anthocyanins was induced by the introduction of *UFGT* regulated by MybA genes, resulted in reddish-purple spots in embryos. The activities and properties of UFGT enzymes were demonstrated related to the high amounts of anthocyanins accumulation in cultivated strawberry (*Fragaria x ananassa*) [49]. A *Citrus paradisi UFGT* gene was cloned and introduced in recombinant expression system to investigate the function of anthocyanin glucosylation [50]. Liu et al. reported the functional characterization of two second *UFGT* genes, *AcUFGT6b* and *AcUFGT7c* from ‘Hongyang’ kiwi- fruit, which cluster with other plant GGTs. 3-O-glycosylated anthocyanins were recognized by the recombinant protein of *AcUFGT6b*, and resulted in the new anthocyanins accumulation under the co-expression of other two genes, *AcMYBF110* and *AcUFGT3a*, also the increased content of cyanidin 3-O-xyl-galactoside was observed after the overexpression of *AcUFGT6b* together with *AcMYBF110* and *AcUFGT6b7c*-RNAi, which showed *AcUFGT6b* was responsible for the end-product of the anthocyanin biosynthesis pathway in red-fleshed kiwifruit [23].

In this study, a striking difference in *PmUFGT3* gene was detected in two Japanese apricot lines. In all populations of Japanese apricot species with green-skinned fruit, *PmUFGT3* is a nonfunctional gene; however, in all populations of Japanese apricot species with red-skinned fruit, *PmUFGT3* is a functional gene. It indicated a relationship between fruit skin color difference and loss of functional *PmUFGT3* gene.

### The key SNP mutation of *PmUFGT3* associated with red pigmentation.

*UFGT*, as the structural genes in the anthocyanin biosynthesis pathway are highly conserved among flowering and fruit species [51, 52]. SNPs have been found as the most common genetic mutations in the genome of eukaryotic species, mutations in structural genes can affect the biosynthesis of anthocyanin, then resulted in diverse phenotypes [24, 53].

One SNP s30 on *UFGT* was associated with the skin and pulp color phenotypes based on RP-HPLC analysis and visual characterization in *Vitis vinifera* [54]. One indel marker of *UFGT* was used to constructed carrot linkage maps controlling the anthocyanin pigmentation [55]. The SNPs of coding region sequence of *UFGT* prompted the development of molecular markers which enabled the discovery of discriminant SNPs (1/34 bp) and the reconstruction of 130 V*.vinifera* distinct genotypes, and infer SNP-based genotypes of grapevine for assessing the genetic identity including different color pigmentation cultivars [56]. A *UFGT* high resolution melting (HRM) assay presents 58 SNPs within 22 grapevine varieties and produced differentiated melting curves for 18 haplotypes which is efficient in grapevine varietal discrimination [57]. Wu et al. found the *UFGT* gene had a significant higher expression in red flower than white flower in Japanese apricot, in addition, there were SNPs resulted nonsynonymous mutations which may affect the enzyme activity [37].

In this study, we found that CDS sequences of *PmUFGT3* showed high similarity between two different-colored skin fruit, but there were 23 SNPs were detected in a genomic cDNA *PmUFGT3* gene comparison between red and green-skinned fruits of Japanese apricot, 16 Transition substitutions were more common than 7 transversions (with a ratio of transitions to transversions close to 2.3:1). among all SNPs, SNPs at positions 201, 660, 789, 894, 1009, 1017, 1050, 1066, 1149, 1336, 1477 were classified as synonymous mutations. For a long time, people have believed that synonymous SNPs are irrelevant, because the main sequence of the protein is preserved [58, 59]. Although synonymous mutations may change the structure, function, and expression levels of proteins strongly associated with CpG islands, several mechanisms are now being elucidated. Studies have shown that synonymous polymorphisms can affect messenger RNA splicing, stability and structure, and protein folding [60, 61]. These changes can have a significant impact on the function of the protein, but we did not detect such a CpG island, and the higher frequency of A-G SNPs transitions occurred, which showed these synonymous mutations show no function associated with anthocyanin pigmentation.

The SNP at 664, 669–670, 764, 1014, 1318–1319, 1332, 1441–1442 bp was classified as nonsense mutations, resulting in 7 amino acid substitutions in red-skinned fruits of Japanese apricot compared with green-skinned. Among codon D222 (AAG) was replaced by CAG (E) for *PmUFGT3-GM1*, codon D224 (ATC) was changed to CTC (L) for *PmUFGT3-GM2*, codon D255 (GAT) was replaced by AAT (N) for *PmUFGT3-GM3*, codon D338 (TCG) was changed to CCG (P) for *PmUFGT3-GM4*, codon D440 (GAC) was replaced by AGC (S) for *PmUFGT3-GM5*, codon D444 (GAG) was changed to GAT (D) for *PmUFGT3-GM6*, and the codon 481 (AAG) was substituted with GCG (A) for *PmUFGT3-GM7*. Which only one *PmUFGT3-GM6* mutagenesis induced the formation of red cells in the skin, the transgenic plant showed complementation of the red-skinned phenotype as *PmUFGT3-R* from red-skinned cultivars “RHM”, determined by visual examination of the skin color (Figs. 8 and 9) while other six site-directed mutagenesis *PmUFGT3* still have the same phenotype as green-skinned cultivars which no red-pigmentation on the fruit skin. In conclusion, the *PmUFGT3-GM6* SNP is a candidate genetic marker for red-skinned color. Obviously, it would be classified as a non-conservative amino acid substitution and might have a major impact on protein function.

**Fig. 11:**
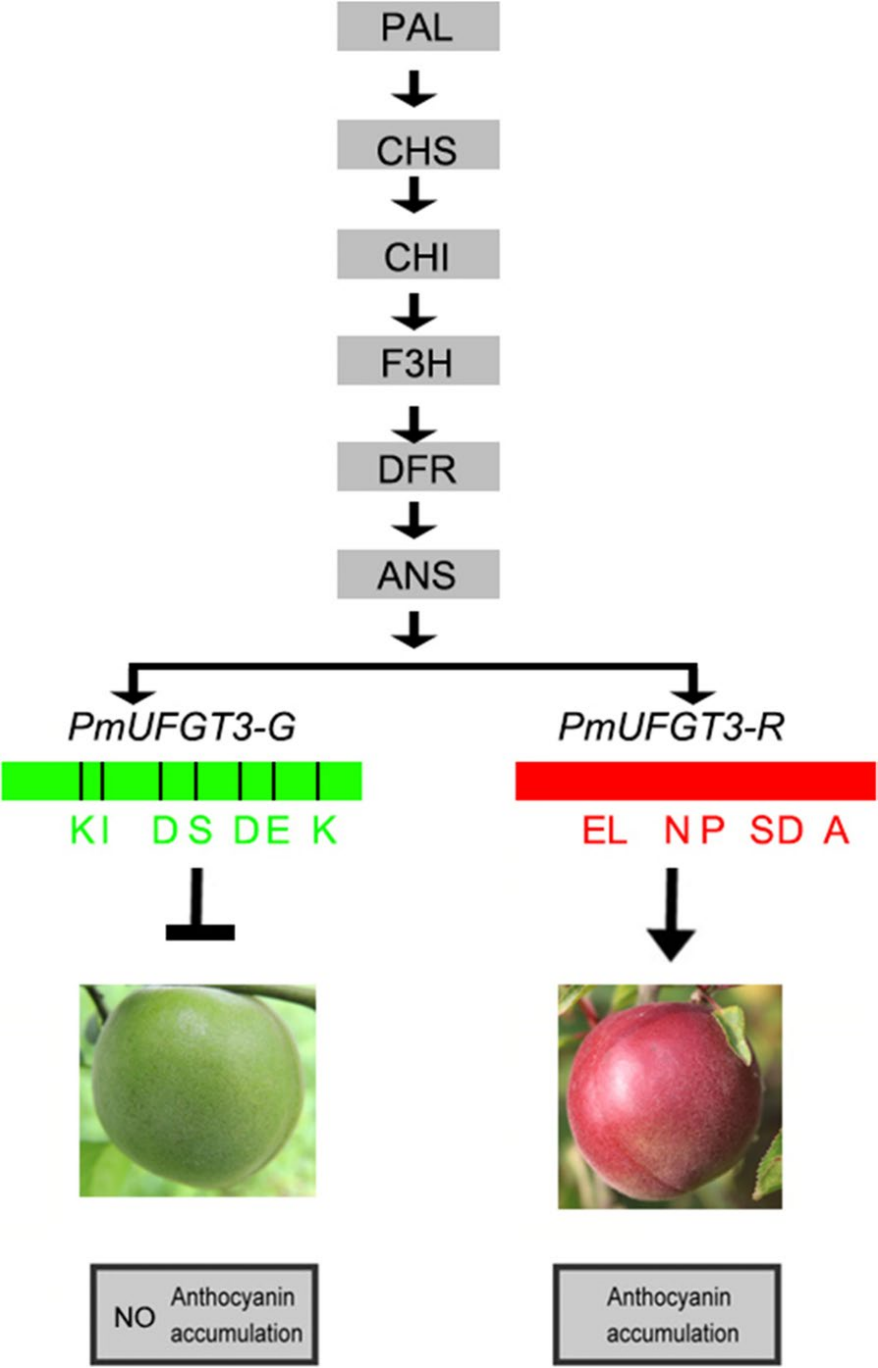
Diagram of anthocyanin metabolic pathways and enzymatic steps regulated by the *PmUFGT3* allele in the different-colored skin fruit in Japanese apricot

Here, we propose a working model for *PmUFGT3* to modify cyanidin 3-O-glucoside in Japanese apricot (Fig. 11). The enzyme *PmUFGT3-R* then attracts the UDP-xylose to the cyanidin 3-O-glucoside generating cyanidin 3-O-xyl-glucoside. While *PmUFGT3-G*, does not have this ability due to its structural variation.

## Conclusions

In this study, gene catalyzing the final step, namely UDP-glucose: flavonoid 3-O-glucosyltransferase (*PmUFGT3*) involved in anthocyanin synthesis were identified from red-skinned of Japanese apricot. Our investigations revealed that *PmUFGT3* is a functional glucosyl-transferase structure–function of anthocyanin synthesis. The fruit color difference between red and green-skinned may be related to the variation of *PmUFGT3* gene. Key point mutation may be one nonsense mutation, Transition between T and G resulting in one amino acid substitution D to E in green-skinned fruits of Japanese apricot compared with red-skinned cultivars, and propose a working model for *PmUFGT3* to modify cyanidin 3-O-glucoside in Japanese apricot.

## Methods

### Plant materials

Six cultivars of Japanese apricot were used in this study and collected from the National Field Gene Bank for *Prunus mume* at the agricultural research station, Nanjing Agricultural University, Nanjing, P.R. China. Based on fruit skin pigmentation, the cultivars were classified into two groups: green-skinned and red-skinned. Green-skinned cultivars included ‘Qingjia No.2’ (QJM), ‘Yanglao’ (YLM) and ‘Shinano koume’ (SKM). The red-skinned cultivars included ‘Ruantiao Hongmei’ (RHM), ‘Xiaoye Zhugan’ (XZM) and ‘Zhonghong’ (ZHM). Each three individual plants with immature green-skinned and red-skinned fruits were collected in May 2017.

Total 44 accessions of Japanese apricot cultivars were used for SNPs validation listed in Table 2 by Sanger sequencing, including a set of 15 diverse genotypes (set A) of different origins, which were identified as red-skinned cultivars group based on morphology, a second set of 29 genotypes (set B) were identified as green-skinned cultivars group.

**Table 2:** Phenotypic and cultivar data for 44 Japanese apricot cultivars used in this study

Phenotype	No	Cultivars	Phenotype	No	Cultivars
Red-skinned	1	Jiuzhongmei	Green-skinned	23	Huaxiangshi
Red-skinned	2	Wanhong	Green-skinned	24	Henghe
Red-skinned	3	Ruantiao Hongmei	Green-skinned	25	Xiyeqing
Red-skinned	4	Xiaoye Zhugan	Green-skinned	26	Yuying
Red-skinned	5	Hongnong	Green-skinned	27	Gucheng
Red-skinned	6	Daqiandi	Green-skinned	28	Pinzimei
Red-skinned	7	Zhonghong	Green-skinned	29	Longyan
Red-skinned	8	Zaohong	Green-skinned	30	Guangdong Huangpi
Red-skinned	9	Dalizhong	Green-skinned	31	Siyuemei
Red-skinned	10	Weishanzhong	Green-skinned	32	Yunnan Hongmei
Red-skinned	11	Hongding	Green-skinned	33	Taoxingmei
Red-skinned	12	Nanhong	Green-skinned	34	Yanglao
Red-skinned	13	Kaidi	Green-skinned	35	Changnong 17
Red-skinned	14	Daye Zhugan	Green-skinned	36	Shuangtaomei
Red-skinned	15	Huangxiaoda	Green-skinned	37	Yanhua
Green-skinned	16	Yeliqing	Green-skinned	38	Xuemei
Green-skinned	17	Sichuan Baimei	Green-skinned	39	Fenban Guomei
Green-skinned	18	Xinnong Xiaomei	Green-skinned	40	Daroumei
Green-skinned	19	Tonglv	Green-skinned	41	Danfenghou
Green-skinned	20	Lve	Green-skinned	42	No.67
Green-skinned	21	Sichuan Qingmei	Green-skinned	43	Qingjia No.2
Green-skinned	22	Yueshijie	Green-skinned	44	Dongqing

### Experimental validation of SNPs

A subset of 30 randomly selected SNPs from genome re-sequencing identified used for validation by sanger sequencing. Primer No.27 were designed to obtain amplicons of 300–1,000 bases containing at least one putative SNP (Additional file 4). Two µl of DNA was amplified with 1 µM of each primer, 0.2 mM dNTP (TaKaRa), 3 mM MgCl_2_ and 2 U Taq DNA polymerase (TaKaRa), using the following cycling program: 5 min at 94 °C, then 40 cycles of 30 s at 94 °C, 90 s at 60 °C, 90 s at 72 °C and final extension 10 min at 72 °C. PCR products were detected by 2% agarose gel electrophoresis and ethidium bromide staining. Purified PCR products were subjected to Sanger sequencing performed by TSINGKE Biological Technology (Nanjing, China). Genotype calling and subsequent SNP calling were examined chromatogram trace files and detect variants by extracting or comparing signals in the peaks of traces, using the NovoSNP software [62].

### Investigation of physiological indicators and agricultural traits

Fruit physiological traits were measured at the same ripening time point (Fig. 1). Vertical height, flank diameter and width were measured with a Vernier calliper (GuangLu, China). Single fruit weight was measured with an electronic balance (METTLER TOLEDO, Switzerland). Pulp from peeled Japanese apricot was squeezed in a cheesecloth, and the filtered, homogenized juice was used for determination of soluble solids using a digital refractometer (ATAGO, Japan).

The colour of all fresh-cut fruits was directly measured with a Minolta CR-400 Chroma Meter (Konica Minolta Sensing, Inc., Osaka, Japan) using the Illuminant D75 and an observation angle of 10°, which had been calibrated with a standard white plate (Y = 94.00, x = 0.3158, y = 0.3322). Three readings of L* (lightness), a* (red-green chromaticity) and b* (yellow-blue chromaticity) coordinates were recorded for each Japanese apricot sunny surface. Each sample chose from 10 replicates by changing the position of the sunny surface at the maturity stage. Chroma and hue angle were calculated as Chroma = (a*2 + b*2)1/2 and Hue = tan − 1(b*/a*) [63].

### Determination of anthocyanin accumulation

Anthocyanin extraction and quantification was performed as previously described [64]. Briefly, 1 g fresh weight (FW) hypocotyl or fruit skin material was transferred into a tube containing 4.3 mL of extraction solution (1-propanol/HCl/distilled water, 18/1/81, v/v/v). The tubes were then placed in boiling water for 6 min and incubated in the dark overnight at room temperature. An additional 3.7 mL of extraction solution was then added to the mixture, the sample was mixed and centrifuged at 1,000 g for 5 min. The supernatant was filtered through a 0.45 μm filter (Millipore), and the amount of anthocyanin in the extracts was quantified using a spectrophotometer by reading at A_535_ and A_650_ and expressed as (A_535_-A_650_) per gram of FW. Each analysis was performed with three biological replicates.

### Isolation and sequencing of the *PmUFGT3* gene

Plant materials were harvested, frozen in liquid nitrogen, and then ground under RNase-free conditions. The RNA was extracted using the TRizol reagent (Invitrogen), following the manufacturer’s instructions, and then treated with DNase I at 37 °C for 30 min. The RNA was then reverse transcribed using the PrimeScript first-strand cDNA synthesis kit (TaKaRa), following the manufacturer’s instructions. A 10-mL aliquot of cDNA was diluted to 100 mL with water, and 2 mL (50 ng) of the diluted cDNA was used for PCR. The *PmUFGT3* PCR product was cloned into pClone007 Blunt Vector (TSINGKE) and confirmed by sequencing the full open reading frame.

Full-length cDNAs of *PmUFGT3* were obtained by RT-PCR using the primer pairs *PmUFGT3-F/R* (Additional file 5). The full-length cDNA sequence was used to search homologous sequences via the National Center for Biotechnology Information BLASTX as previously described [65]. Both the theoretical isoelectric point and molecular weight were calculated online (http://www.expasy.ch/tools/pi_tool.html). The ORF of the full-length *PmUFGT3* was identified via Genscan (http://genes.Mit.edu/GENSCAN.html) and motifs were found with Plantcare (http://bioinformatics.psb.ugent.be/webtools/plantcare/html/). The secondary structure of the predicted *PmUFGT3* protein was constructed with the Predict Protein tool (http://www.predictprotein.org/) and the three-dimensional structure of the predicted *PmUFGT3* was modeled via SWISS-MODEL with Automated model (http:// swissmodel.expasy.org/). A phylogenetic analysis of the *PmUFGT3* protein was performed based on the deduced protein sequence using the NJ method, which was implemented in MEGA 5.0.

### Sequence alignment and phylogenetic analysis

The amino acid sequences of *PmUFGT3*, obtained from full-length cDNAs, were aligned using the MEGA v 5.05 and ClustalW software as previously described [66]. Alignment parameters (gap opening penalty and gap extension penalty) used were 10.00 and 0.1 for pair-wise alignments, and 15.00 and 0.30 for multiple alignments. A phylogenetic tree was constructed and visualized using the neighbor-joining (NJ) method in MEGA v 5.05. The statistical significance of individual nodes was assessed by bootstrap analyses with 1,000 replicates.

### Construction of the plant expression vector and transient transformation of Japanese apricot fruit skins

To generate the 35S:UFGT3 construct, the coding regions of *PmUFGT3* were cloned into the pCAMBIA1301 binary vector driven by the CaMV 35S promoter. The binary Ti vector pCAMBIA1301*-PmUFGT3*-35SN was used for transformation. It was constructed as follow. The cauliflower mosaic-virus (CaMV) 35S promoter and nopaline synthase terminator region were added into the *pCambia1301* vector, the binary Ti vector pCAMBIA1301-35SN contains Kanamycin resistance for screening transgenic lines. After being digested with XbaI and SacI restriction enzymes, the cDNA of *PmUFGT3* was inserted between the CaMV35S promoter and nopaline synthase terminator region of the Ti vector pCAMBIA1301-35SN, which has been digested with the same enzymes. The new construct, named pCAMBIA1301-*PmUFGT3*-35SN, was electroporated into agrobacterium tumefaciens strain GV3101. The positive clones were selected on LB plates containing 50 mg^.^L^−1^ kanamycin and identified by PCR amplification using *PmUFGT3* specific primers. Separate strains containing *PmUFGT3* and GUS fused to the 35S promoter in the pCAMBIA1301 vector were infiltrated or co-infiltrated into the abaxial fruit surface. Each infiltration was performed using three fruits from the same plants. Photographs were taken 48 h after infiltration.

### Transient transformation analysis in Arabidopsis mutant line *Atufgt*

Seeds of wild-type (Col-0) *A. thaliana* and overexpression lines (pCAMBIA1301-*PmUFGT3*-35SN) in the Col-0 background were grown on 1/2 MS medium as previously described [67]. The mutant line *Atufgt* (*ugt78d2)* were obtained from the European Arabidopsis Stock Centre (uNASC). Seeds were subjected to a chilling treatment at 4 °C for 72 h and then transferred to white light at 24 °C under long-day conditions (16-h light/8-h dark). Five-day-old Arabidopsis seedlings were used for hypocotyl measurements. At least 10 seedlings were imaged, and hypocotyl color were measured using ImageJ software (https://imagej.nih.gov/ij/).

### Site-directed mutagenesis of *green-skinned PmUFGT3-G* mutant genes

Site-directed mutagenesis was performed with the Easy Mutagenesis System (TransGen Biotech Ltd. Co., Beijing, China) and TransStart FastPfu DNA polymerase as previously described [68]. The mutated plasmid pCAMBIA1301-*PmUFGT3*-*G* template (methylated plasmid template) from green-skinned cultivars ‘QJM’ can be degraded by DMT digestive enzymes in vitro and DMT-competent cell in vivo, thereby screen performed effectively.

PCR was performed using plasmid Blunt-pCAMBIA1301-*PmUFGT3-G* as the template with two mutant primers for each reaction. Each mutated gene was linked to the surface display plasmid pKFS to form a fused gene with FS, and derived plasmids were named pKFSRm. E. coli *DH5a* strain was used as the host for propagation of plasmids containing mutated RML genes (pKFSRm). All plasmids were verified by Sanger sequencing by Sango (Shanghai, China).

### GUS assays

To detect GUS activity in the calluses, histochemical staining was performed as described previously [69]. The callus tissue was immersed in GUS staining buffer (1 mM 5-bromo-4-chloro-3-indolyl-b-glucuronic acid solution in 100 mM sodium phosphate p pH 7.0, 0.1 mm EDTA, 0.5 mm ferricyanide, 0.5 mm ferrocyanide and 0.1% Triton X-100), at 37C. After staining, the calluses were photographed.

### Statistical analysis

Genetic diversity of the whole collection was analyzed by calculating the observed number of alleles (*Na*), the effective number of alleles (*Ne*), Shannon’s information index (*I*) [70] for each type of markers using POPGEN v.1.32 [71]. Nei’s genetic diversity (*He*), gene flow (*Nm*), fixation index (*Fst*) and polymorphism information content (*PIC*) were calculated using PowerMarker 3.25 [72].

Analysis of variance (ANOVA) was performed to compare cultivar mean values using IBM SPSS Statistics 18 (SPSS Inc., Chicago, IL, USA). The least significant difference test was employed to determine differences between means at a 5% significance level. GraphPad Prism version 6.0 (GraphPad Software. San Diego, CA) was used for graph plotting.

## Supplementary Information

**Additional file 1.** Domains of protein encoded by *PmUFGT3*.

**Additional file 2.** Secondary structure prediction of protein encoded by *PmUFGT3*.

**Additional file 3.** Site-directed mutagenesis information of *PmUFGT3*.

**Additional file 4.** Information of primers for No.27 SNP genotyping.

**Additional file 5.** Primer sequence of *PmUFGT3* overexpression vector. 

## Data Availability

Accession codes: The full length of the *PmUFGT3* gene sequence in this paper had uploaded to the GenBank (Submission ID is 2596019) and the accession numbers will be supplied once they are available. The BioProject is PRJNA371370. The sequence data for the ‘XZM’ cultivar has been deposited at BioSample under accession SAMN06298250. The sequence data for the ‘ZHM’ cultivar have been deposited at BioSample under accession SAMN06298251. The sequence data for the ‘SKM’ cultivar have been deposited at BioSample under accession SAMN06298252. The sequence data for the ‘YLM’ cultivar have been deposited at BioSample under accession SAMN06298253. The sequence data for the ‘RHM’ cultivar have been deposited at BioSample under accession SAMN06298254. The sequence data for the ‘QJM’ cultivar have been deposited at BioSample under accession SAMN06298255.

## Abbreviations

C4H: Cinnamic acid 4-hydroxylase
PAL: Phenylalanine ammonia-lyase
4CL: 4-Coumarate-CoA ligase
CHI: Chalcone isomerase
CHS: Chalcone synthase
DFR: Dihydroflavonol 4-reductase
F3H: Flavanone 3-hydroxylase
F3’H: Flavanone 3’-hydroxylase
LDOX: Leucoanthocyanidin dioxygenase
UFGT: UDP-glucose flavonoid- 3-O-glycosyltransferase
*Cfi*: Chalcone flavonone isomerase
SNPs: Single nucleotide polymorphisms
AFLP: Amplified fragment length polymorphism
GBS: Genotyping-by-sequencing
GWAS: Genome-wide association study
QTL: Quantitative trait locus
*I*: Shannon’s information index
*He*: Nei’s gene diversity
*Nm*: Gene flow
*Fst*: Fixation index
*PIC*: Polymorphic information content
QJM: Qingjia No.2
YLM: Yanglao
SKM: Shinano Koume
RHM: Ruantiao Hongmei
XZM: Xiaoye Zhugan
ZHM: Zhonghong
qRT-PCR: Reverse transcription-quantitative polymerase chain reaction
