# Supplementary figures and images for: Mutations in PmUFGT3 contribute to color variation of fruit skin in Japanese apricot (Prunus mume Sieb. et Zucc.)
Source: BMC Plant Biol. 2022 Jun 24;22:304. doi: 10.1186/s12870-022-03693-8 (PMC9229503; doi:10.1186/s12870-022-03693-8)

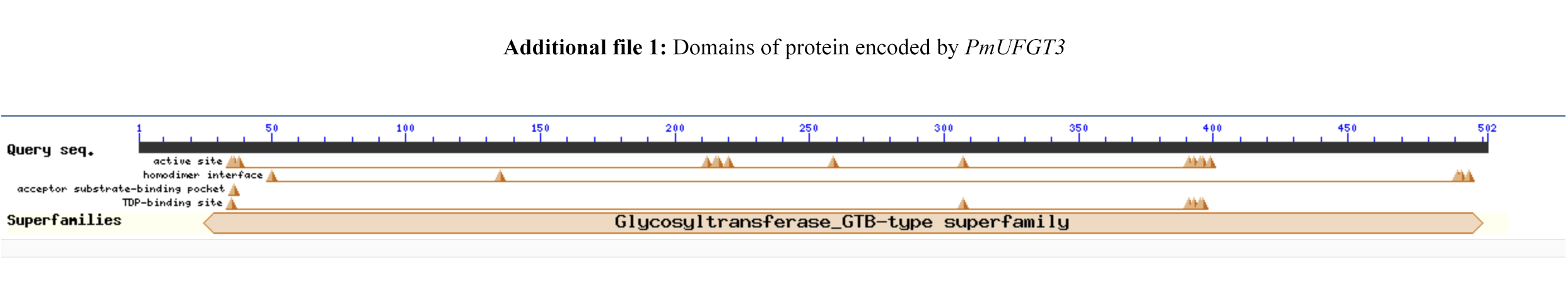

Supplement: Supplementary file 1 — Additional file 1. Domains of protein encoded by PmUFGT3. [file 12870_2022_3693_MOESM1_ESM.tif]

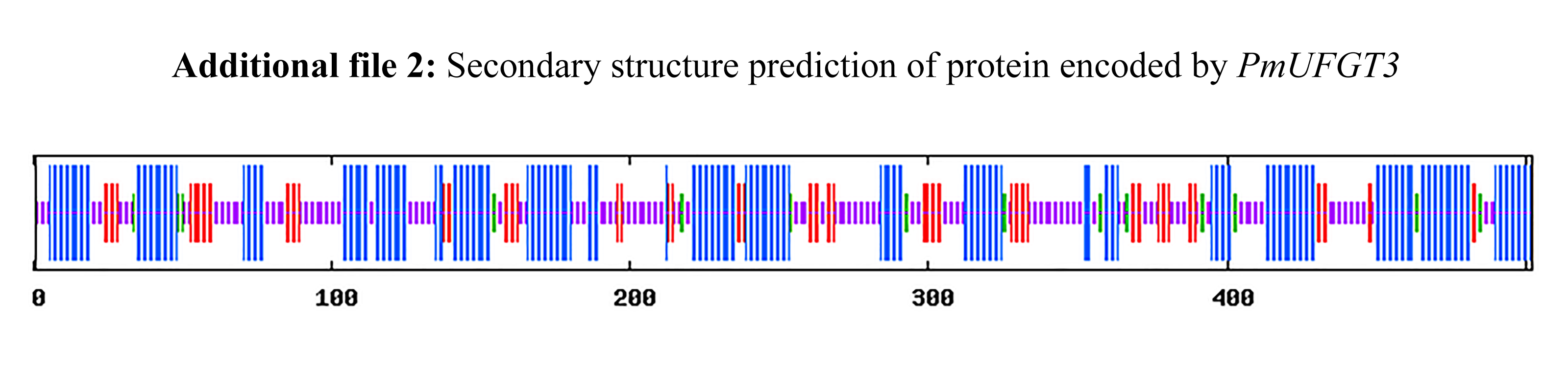

Supplement: Supplementary file 2 — Additional file 2. Secondary structure prediction of protein encoded by PmUFGT3. [file 12870_2022_3693_MOESM2_ESM.tiff]
